# Anatomical Anal Stenosis after PPH: Insights from a Retrospective Study and Rat Model

**DOI:** 10.3390/ijms25063543

**Published:** 2024-03-21

**Authors:** Chia-Cheng Wen, Shih-Ming Huang, Yi-Wen Wang

**Affiliations:** 1 Graduate Institute of Medical Sciences, National Defense Medical Center, Taipei 114, Taiwan; wjason@mail2000.com.tw (C.-C.W.); shihming@ndmctsgh.edu.tw (S.-M.H.); 2 Division of Colon and Rectal Surgery, Department of Surgery, Tri-Service General Hospital, National Defense Medical Center, Taipei 114, Taiwan; 3 Department of Biochemistry, National Defense Medical Center, Taipei 114, Taiwan; 4 Department of Biology and Anatomy, National Defense Medical Center, Taipei 114, Taiwan

**Keywords:** a procedure for prolapse and hemorrhoids, tension force, proinflammatory factors, angiogenic factors, fibrotic factors

## Abstract

High-grade hemorrhoids are usually recommended to receive operational treatments. However, these traditional surgeries are associated with severe postoperative pain. A procedure for prolapse and hemorrhoids (PPH), a circular staple device, has been developed to improve short-term outcomes, including reducing the severity of postoperative pain. PPH, compared to conventional surgery, has been associated with the incidence of anatomical anal stenosis. The causes of stenosis after PPH are not yet clear. We first analyzed the complications of our patients with PPH, and then developed a rat model to verify the tension force of PPH using Hematoxylin-eosin, Masson’s trichrome, immunohistochemistry, and immunofluorescence staining. Our clinical data showed that PPH significantly improved postoperative pain, but that it resulted in higher incidences of complications, including anal stenosis, than hemorrhoidectomy. We simulated the status of PPH and developed a rat model to verify PPH’s tension force, including the scarring area and the deposition of proinflammatory factors, angiogenic factors, and fibrotic factors. The tension wound histological data showed more extensive granulation tissue and inflammatory cell infiltration and a thicker epidermis than the control group on day 12 post-operation and tension treatment. In addition to IL-1β and IL-10 cytokines on day 3 and IL-1β, IL-6, and IL-10 cytokines on day 12 post-operation in the tension group, two angiogenic factors, CD31 and VEGF-A, were found to have a more significant expression on day 7 post-operation in the tension group. The mean scar area was larger and the distribution of fibrotic proteins (collagen 1, α-SMA, CTGF, and MMP2) in the tension group was significantly broader than in the control on day 12 post-operation and tension treatment. Based on the findings of our animal model, the development of a lesser tensile force for PPH to decrease the deposition of proinflammatory factors, angiogenic factors, and fibrotic factors is urgently required.

## 1. Introduction

In the process of skin wound healing, the skin is intentionally designed to maintain its integrity through internal and external forces. The skin is a tensegrity structure that relies on opposing intrinsic forces, and it can also be affected by extrinsic forces like compressive, tensile, and shear forces. Understanding these factors can help with the healing process and promote better outcomes [1,2,3,4]. After receiving a wound, the skin loses its tensegrity, and will experience extrinsic forces. This is due to the opening of the wound and contracture at the wound site, which can result in tensile forces. Myofibroblasts play a key role in the wound contracture, as they can contract and pull the extracellular matrix (ECM) through cell traction forces. As the ECM is shortened, myofibroblasts synthesize new ECM to occupy the space left open by the wound. Understanding these processes can help promote better healing outcomes [3,4,5]. The healing process after a wound is a complex process that involves various factors. One of these factors is the activation of signaling pathways, which are triggered by the mechanical forces acting on mechanoreceptors, such as integrins, G-protein-coupled receptors, ion channels, or growth factor receptors. The healing process after a wound involves a complex interplay of factors, including myofibroblasts and the extracellular matrix [6]. Excessive deposition of ECM can lead to fibrosis, scarring, and loss of tissue function. This is why it is important to monitor the healing process carefully and seek medical attention if necessary.

Hemorrhoids are a condition of anal bleeding during defecation, with or without prolapsing anal tissue [7]. The three main highly vascular cushions, the left lateral, right anterior, and right posterior positions, in the anal canal are rich in blood vessels and muscular fibers to force anal closure with tension [8,9,10]. High-grade hemorrhoids are usually recommended to undergo operational treatments [7]. The closed (Ferguson) hemorrhoidectomy and open (Milligan–Morgan) hemorrhoidectomy are the most used and effective surgeries. However, these traditional surgeries are associated with severe postoperative pain. The literature has also demonstrated that anal stenosis is the most troublesome complication after hemorrhoidectomy, and the possible reason for stenosis is contracting and scarring in the anal canal during the wound healing process [11,12]. When our skin becomes injured, our body automatically starts a series of events known as the “cascade of healing” to repair the damaged tissues. This cascade is split into four overlapping phases: Hemostasis, Inflammatory, Proliferative, and Maturation. The healing process is remarkable and intricate, but it is also vulnerable to interruption due to various factors, such as moisture, infection, and maceration. Age, nutritional status, and body type are systemic factors that can also affect the healing process. However, when the right healing environment is created, the body can excel at replacing and healing devitalized tissue.

During the proliferative phase of healing, contraction occurs and plays a crucial role in closing wounds. This process involves the formation of a cell-rich granulation tissue. While contraction is important for wound closure, it can also have negative effects if it becomes excessive. If the contraction is too strong, it can lead to undesirable contracture and scarring, which can cause cosmetic and functional problems [13]. Myofibroblasts play an important role in wound closure, generating contractile forces that help bring the edges of an open wound together. However, excessive myofibroblast activity can lead to tissue fibrosis and scar formation. The right healing environment can prevent these issues from occurring.

The procedure for prolapse and hemorrhoids (PPH), a circular staple device, was developed to improve short-term treatment outcomes [7]. PPH was invented to treat mixed hemorrhoids patients, with the aim of promoting the reduction of the anal cushion by resecting the submucosal tissue of the lower rectum and anastomosing the broken end of the mucosa. However, negative effects of PPH have been reported, and the recurrence rate of prolapsed hemorrhoids is high. Three systematic review articles demonstrated that PPH, compared to conventional surgery, has a higher risk for poor long-term outcomes and increased incidence of anal stenosis [14,15,16]. A recent study of network meta-analysis demonstrated that PPH is best at reducing postoperative anal stricture and suggests that PPH has the least anal stenosis [17]. A study demonstrated excised specimens not only include the mucosa and submucosa, but also the anal sphincter [18]. Young Ki Hong et al. [19] reported the resected doughnuts in 98.4% of patients had muscle fibers, and smooth muscle was found in the resection of one patient. The work of Vysloužil reported increased anal sphincter tension persisted 6 months after hemorrhoidectomy [20]. Therefore, the bilateral side of the wound might be stretched in a high-tension environment, while accompanying tensile forces slow the process of wound healing. Hence, there is an unmet medical issue regarding the incidence rate and mechanism of anal stenosis in PPH.

The principle of PPH is an interruption of the blood flow to the hemorrhoids by circumferentially excising the mucosa and submucosa. Anal stenosis is a severe complication after PPH. If a patient’s stenosis condition becomes worse, another treatment may need to be considered. The causes of stenosis after the operation remain unclear, but one of the potential causes is that the stapled ring is placed adjacent to active cells, which react by scarring and shrinking [21]. The anatomical anal stenosis is related to increased fibrous scar tissue formation, which disables stretching of the anal canal via the narrowing of the anal sphincter. In this study, we tried to address the unmet medical issue of the incidence rate and mechanism of anal stenosis in PPH. Therefore, we first analyzed the complications of our patients with PPH, and then developed an animal model to verify whether the tension force of PPH was involved in the anatomical anal stenosis, and further explored the corresponding mechanisms involved in the deposition of proinflammatory factors, angiogenic factors, as well as fibrotic factors. We hope that this study might provide information relevant to overcoming the complication of anatomical anal stenosis.

## 2. Results

### 2.1. The Definition and Analysis of the Tension of the Operation Wound in Hemorrhoids

**Figure 1:**
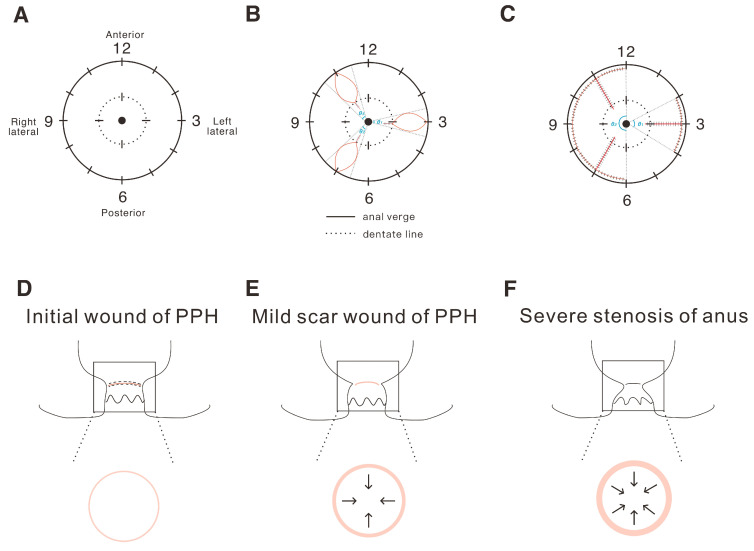
Carton schematic of the anal clock to demonstrate how to define the wound range ratio in anus surgery and the progression of anal stenosis after the PPH surgery. (**A**) The normal condition of the anus; (**B**) the open (Milligan–Morgan) hemorrhoidectomy; (**C**) the closed (Ferguson) hemorrhoidectomy. θ represents the angle of the surgical wound extent in a column. (**D**) Initially, after the PPH surgery, the wound showed normal wound tension. (**E**) During wound healing, the tension of the ring wound increased slightly toward the inner anus, and then a slight scar was formed, while the patient’s defecation function was not affected. (**F**) Finally, as the wound healing gradually progressed, the wound tension continued to increase, leading to hypertrophic scars, and finally severe anal stenosis.

First, we designed a carton schematic view to demonstrate how to define the wound range ratio in the anus surgery. In Figure 1A, the anal clock shows the normal condition of the anus, with the completely anal verge and dentate line. Figure 1B shows the open (Milligan–Morgan) hemorrhoidectomy, and we could then calculate the total wound ratio (%) as the sum of the affected portion by θ1 + θ2 + θ3/360 × 100%. In Figure 1C, showing the closed (Ferguson) hemorrhoidectomy, the wound ratio (%) was the sum of the affected portion, calculated by θ1 + θ2/360 × 100%. The wound range ratio (%) of the anus was defined as the ratio of the circumferential wound of the anus (CWA). Figure 1D–F gives a schematic diagram to show the relationship between wound tension and anal stenosis. An atypical increase in wound tension will lead to the proliferation of scars and to severe anal stenosis, and subsequently affect the defecation function.

Our recent retrospective study analyzed 125 cases of conventional hemorrhoidectomy performed from January 2012 to December 2017. The data from the Tri-Service General Hospital (TSGH) indicated that the larger the wound ratio (CWA%) in the anal surgery, the higher the painkiller consumption, hospital stay, and pain during defecation tended to be. Our other retrospective study analyzed 174 cases from January 2011 to December 2017, comparing hemorrhoidectomy and pure PPH. Complications such as anatomical anal stenosis were significantly higher in the PPH group compared to the hemorrhoidectomy group. A total of 75 patients were recruited to the PPH group, and 99 patients to the hemorrhoidectomy group (Table 1). Between these two groups, the mean age of the patients was 51.01 ± 12.28 years in the PPH population and 51.87 ± 15.95 years in the hemorrhoidectomy group. The percentages of female patients in each group were 48% and 46.5%. Other demographic data also showed no significant differences between the two groups in Grade IV, ASA, and constipation history.

According to surgery complexity, the data showed the average operation time in the PPH group was significantly shorter than in the hemorrhoidectomy group (41.60 ± 13.59 min vs. 51.72 ± 17.85 min). However, there was no significant difference in the length of hospital stay and recurrence. For post-operational pain relief, the number of painkiller administrations in the PPH group was obviously lower than in the other group (*p* = 0.002). Regarding overall compliance after surgery, the incidence rate in the PPH group was higher than in the hemorrhoidectomy group (*p* = 0.042). Similarly, in the subgroup of patients with anatomical anal stenosis, the incidence in the PPH group was 5, and no anatomical anal stenosis occurred in the hemorrhoidectomy group, a significant difference between the two groups (*p* = 0.032). No significant difference was found in the rest of the complications.

We further implemented multiple logistic regression analysis models to identify factors associated with the overall complications. Table 2 shows that patients in the PPH group (OR = 5.913, 95% CI: 4.903–40.541, *p* = 0.049) and longer hospital stays (OR = 2.827, 95% CI: 1.615–9.420, *p* = 0.016) were more likely to be associated with higher incidence rates of complications.

### 2.2. The Verification and Effects of Tensile Force on Wounds in Our Animal Model

**Figure 2:**
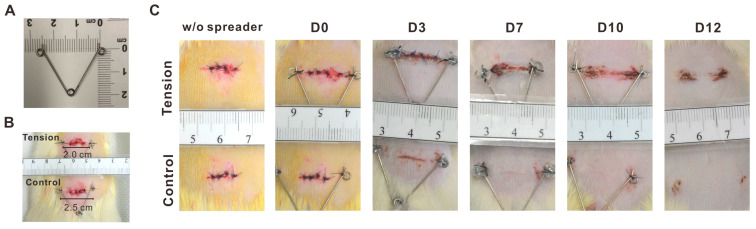
The tool and quantifiable tension applied in this study. (**A**) Picture of the spreader, the distance of both ends was 2.5 cm in a relaxed state. (**B**) In the tension group, the spreader pressed both ends to a distance of 2 cm and was fixed to the incision wound, producing a tension of about 85 gm. In the control group, the spreader was fixed to the incision wound without pressing both ends. (**C**) The appearance of linear wound healing progress from day 0 (D0) to day 12 (D12) in the tension and control groups.

In animals such as pigs or dogs, although the anus size and structure are closer to that of human beings, it is difficult to create a consistent tension at the anus of these animals because it can impact their movement and excretion, leading to pain and severe discomfort. Therefore, an animal model was designed using a spreader with adjustable tension according to its opened size, fixed to the back of Sprague Dawley rats. A sutured wound was created on the back of the rat (Figure 2A,B) to simulate the wound environment after anal surgery, and quantifiable tension was applied to observe the effects of tension on scar hyperplasia during wound healing.

The appearance of the linear wound healing progress in the two groups from day 0 to day 12 is shown in Figure 2C. On day 1, inflammation was marked with oozing of blood in both groups. On Day 3, the wound started to heal in the control group. On Day 7, the linear wound healing in the tension group still showed scabbing. In contrast, the wound of the control group was entirely closed and without a scab. On Day 10, the recovered site swelled and reddened in the tension group, indicating scar formation. On Day 12, the skin became knurled, and a scab still existed in the tension group, while the skin was smooth in the control group.

**Figure 3:**
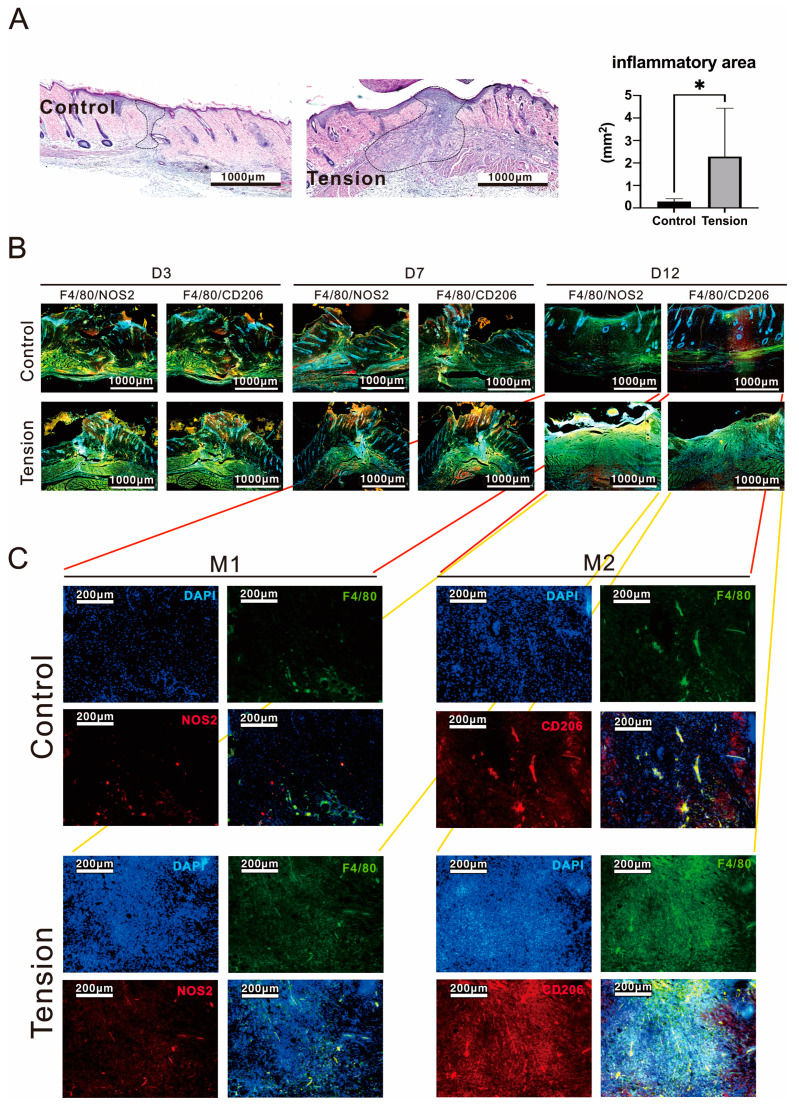
Histological images of inflammatory cell infiltration in the control and tension groups. (**A**) H&E staining, quantitative data of the inflammatory cell infiltration area. Data are presented as the mean ± standard deviation (n = 8; * *p* < 0.05). (**B**,**C**) M1 and M2 cell distribution of the granulation tissue on days 3, 7, and 12. The data of (**C**) were further analyzed from the (**B**) D12 data (red lines for control group and yellow lines for tension group).

**Figure 4:**
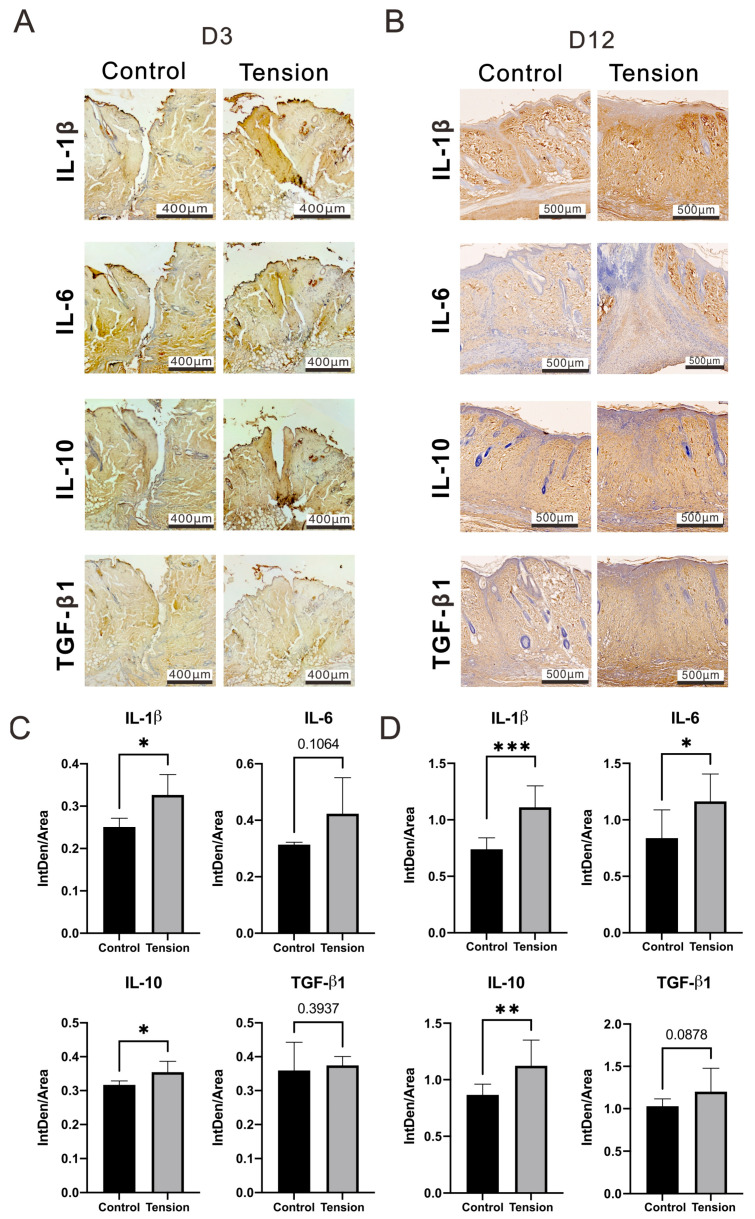
Inflammatory-related cytokines in the control and tension groups. Immunohistochemistry staining and relative quantitative data of IL-1β, IL-6, IL-10, and TGF-β1 in the control and tension groups on day 3 (**A**,**C**) and day 12 (**B**,**D**) after the operation. Scale bar = 400 μm (**D**) and 500 μm. Data of integrated density of staining within a defined area (IntDen/Area) are presented as the mean ± standard deviation (n = 3 at D3, n = 8 at D12; * *p* < 0.05, ** *p* < 0.01, *** *p* < 0.0001).

The cascade of healing was divided into four overlapping phases: Hemostasis, Inflammatory, Proliferative, and Maturation. We analyzed proteins related to the Inflammatory, Proliferative, and Maturation phases at day 3 (D3), 7 (D7), and 12 (D12), respectively, in the wound areas. We first examined the cell types in the wound area using H&E staining analysis. We further examined the M1 and M2 macrophage population and which were increased at D3, D7, and D12. Our analytic data showed that the tension wound histological data had more extensive inflammatory cell infiltration (2.28 ± 1.97 mm^2^ vs. 0.28 ± 0.12 mm^2^, *p* = 0.034) and a thicker epidermis than the control group on D12 post-operation and tension treatment (Figure 3A), since the M1 and M2 macrophages were decreased in the control group but not in the tension group at D12 (Figure 3B). Both the M1 and M2 macrophages were dramatically increased in the tension group on D12 post-operation and tension treatment (Figure 3C). Based on inflammatory cell infiltration and new blood vessel formation in the tension group, we checked the macrophage differentiation and cytokine or growth factor of inflammation and angiogenesis in different healing phases. Some inflammatory-related cytokines, including IL-1β, IL-6, and IL-10, were analyzed and they were more elevated in the wound site of the tension group than in the control group on D3 and D12 post-operations with the tension treatment. Our D3 IHC data showed that significant increases in IL-1β (0.33 ± 0.04 vs. 0.25 ± 0.02, *p* = 0.0216) and IL-10 (0.35 ± 0.03 vs. 0.32 ± 0.01, *p* = 0.0445) proteins occurred in the tension group (Figure 4A,C). However, the increased amount of IL-6 (0.42 ± 0.10 vs. 0.31 ± 0.01, *p* = 0.1064) protein was not significant in the tension group. In the wound healing process, TGF-β1 plays a role in the regulation of re-epithelialization, as well as processes such as inflammation, angiogenesis, and the formation of granulation tissue [22]. However, our IHC data showed no difference in TGF-β1 expression in the two groups (0.37 ± 0.02 vs. 0.36 ± 0.07, *p* = 0.3937) on D3 post-operation and tension treatment. Our D12 IHC data show that significant increases in IL-1β (1.11 ± 0.18 vs. 0.74 ± 0.09, *p* = 0.00008), IL-6 (1.16 ± 0.22 vs. 0.84 ± 0.23, *p* = 0.0032), and IL-10 (1.12 ± 0.21 vs. 0.87 ± 0.09, *p* = 0.0084) proteins occurred in the tension group (Figure 4B,D). M2 macrophages play a significant role in secreting TGF-β and its implications in immune responses and tissue remodeling [23,24]. Therefore, the increasing trend of TGF-β (1.2 ± 0.25 vs. 1.03 ± 0.08, *p* = 0.0878) (Figure 4B) in the tension group might be related to the increase in M2 macrophages, as indicated by the data shown in Figure 3B,C.

**Figure 5:**
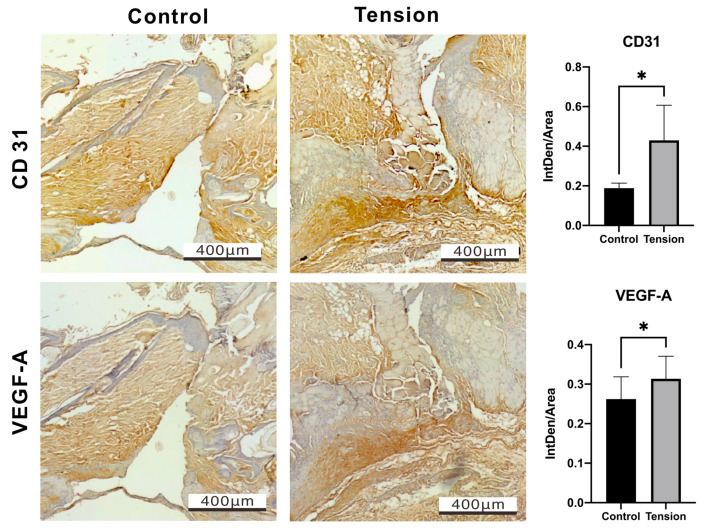
Immunohistochemistry staining and relative quantitative data of CD31 and VEGF-A in the tension and control groups on day 7 after the operation. Scale bar = 400 μm. Data of integrated density of staining within a defined area (IntDen/Area) are presented as the mean ± standard deviation (n = 3; * *p* < 0.05).

The Proliferative phase features the development of granulation tissue, neovascularization, and re-epithelialization. Granulation tissue fills the wound bed with connective tissue throughout this stage while, simultaneously, new blood vessels form [25]. Hence, we further examined whether the new blood vessels were affected by the tension force via the expression of angiogenic factors, including CD31 and VEGF-A (Figure 5). Our IHC data showed a significant increase in CD31 (0.43 ± 0.14 vs. 0.19 ± 0.08, *p* = 0.0396) and VEGF-A (0.31 ± 0.05 vs. 0.26 ± 0.05, *p* = 0.0232) proteins on D7 post-operation in the tension group compared to the control group, supporting the idea that inflammation can promote angiogenesis.

**Figure 6:**
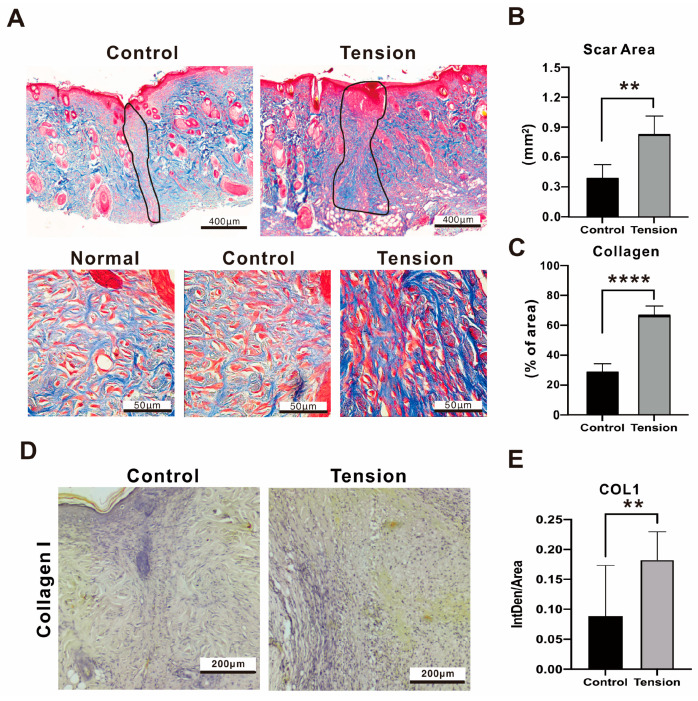
Histological analysis of scar tissue from the tension and control groups on day 12 after the operation. (**A**) Masson’s trichrome staining, (**B**) the scar area, (**C**) deposition percentage of collagen fiber quantitative data (% of area), (**D**,**E**) immunohistochemistry staining and relative quantitative data of type one collagen. Data of integrated density of staining within a defined area (IntDen/Area) are presented as the mean ± standard deviation (n = 8; ** *p* < 0.01, **** *p* < 0.0001).

During the remodeling phase, the newly formed tissue gradually acquires strength and flexibility. Excessive myofibroblast activity, characterized by heightened contraction and an excessive production of extracellular matrix (ECM), is widely recognized as a primary factor contributing to tissue fibrosis and the formation of scars [13]. Masson’s trichrome stain is a histological staining method; most formulas stain keratin and muscle fibers red, while collagen and bone are stained blue or green, the cytoplasm light red or pink, and the nuclei dark brown or black. Under the microscope view, the amount of keratin was over-expressed in the D12 tension environment. In addition, the black boundary line indicated the area of the scarring in the tension group was greater than in the control group (0.83 ± 0.47 mm^2^ vs. 0.38 ± 0.36 mm^2^, *p* = 0.002), and the data showed collagen sediments were significantly deposited in the tension group (Figure 6A–C). The collagen deposition (66.06 ± 16.77% of Area vs. 28.08 ± 14.51% of Area, *p* = 0.0001) and type 1 collagen protein expression (0.1829 ± 0.0439 vs. 0.08835 ± 0.08, *p* = 0.0049) in the tension group were significantly higher than in the control (Figure 6D,E).

**Figure 7:**
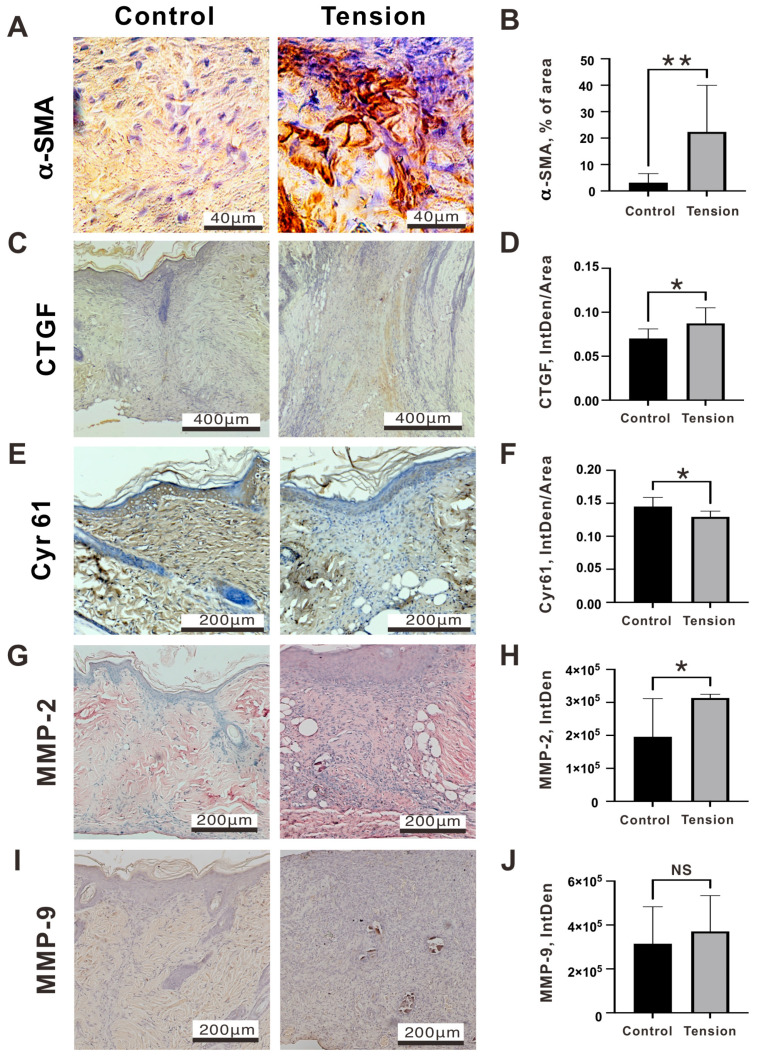
Immunohistochemistry staining and relative quantitative data of (**A**,**B**) α-SMA, (**C**,**D**) CTCF, (**E**,**F**) cyr61, (**G**,**H**) MMP-2, and (**I**,**J**) MMP-9 in the control and tension groups on day 12 after the operation. Data are presented as the mean ± standard deviation (n = 8; * *p* < 0.05, ** *p* < 0.01, NS: not significant).

Fibroblasts exhibit sensitivity to external mechanical forces, leading to the activation of various fibrotic genes. These genes encode proteins like alpha smooth muscle actin (α-SMA) and type I collagen, and their upregulation is mediated by diverse mechanoreceptors [26]. In addition, the cellular contractile forces in the activated myofibroblasts are critical to maintaining scar contracture through their adhesion to the ECM, including cysteine-rich 61 (cyr61/CCN1), connective tissue growth factor (CTGF/CCN2), MMP-2, and MMP-9 [27]. Tensile forces have been shown to drive fibroblasts to a more “synthetic” phenotype through an increase in the expression of α-SMA [28]. The α-SMA deposition in the tension group was significantly higher than in the control group (22.36 ± 16.30% of Area vs. 3.13 ± 3.17% of Area, *p* = 0.019) (Figure 7A,B). GTGF plays a pivotal role as a signaling and regulatory molecule in various biological processes, including but not limited to cell proliferation, angiogenesis, and wound healing [29]. Cyr61 and CTGF could also synergize with growth factors and cytokines to facilitate interactions with the ECM [30]. As myofibroblasts proliferate, an accumulation of Cyr61 within the granulation tissue occurs. This accumulation reaches a critical threshold gradually, prompting the myofibroblasts to enter a state of senescence [31]. In this state, the myofibroblasts stop proliferating and express enzymes that degrade the surrounding matrix, reducing the risk of scar formation [32]. The expression of the CTGF protein in the tension group was higher than in the control group, particularly in the scar area, and there was a statistical significance (0.087 ± 0.02 vs. 0.07 ± 0.01, *p* = 0.0178) (Figure 7C,D), while Cyr61 (0.13 ± 0.01 vs. 0.15 ± 0.01, *p* = 0.0205) was lower than in the control group (Figure 7E,F). In the analysis of ECM degradation enzymes, MMP2 (313,430 ± 9761 vs. 195,909 ± 100,130, *p* = 0.0447) in the tension group was significantly higher than in the control group (Figure 7G,H); however, MMP9 expression (370,975 ± 140,827 vs. 314,559 ± 146,288, *p* = 0.1615) was not significantly different in both groups (Figure 7I,J). In summary, fibrosis-related protein expressions of Collagen 1 (Figure 6D,E), α-SMA (Figure 7A,B), CTGF (Figure 7C,D), and MMP2 (Figure 7G,H) were significantly higher while, in contrast, anti-fibrotic protein Cyr61 was significantly lower on D12 post-operation in the tension group (Figure 7E,F).

## 3. Discussion

The conventional technique of Milligan–Morgan hemorrhoidectomy (MMH) is a widely accepted surgical procedure used to treat hemorrhoids [33]. However, the current guidelines and a meta-analysis both showed a noticeable discrepancy between MMH and other procedures regarding the likelihood of complications and postoperative pain [34]. PPH is another treatment option for internal hemorrhoids and may result in less pain and shorter hospital stays than MMH. However, there have been reports of postoperative complications and adverse events associated with PPH, including anatomical anal stenosis and anal incontinence [14,15,16]. The incidence of anal stenosis after PPH and MMH reported in the literature are 0.2–7.5 and 2.6%, respectively. Our current PPH data were 6.7% compared with no case in Hemorrhoidectomy. However, the latest study of network meta-analysis demonstrated that PPH is best at reducing postoperative anal stricture and suggests that PPH has the least anal stenosis [17]. Compared with this inconsistent analysis, there are many possibilities to explain the current different conclusions in the incidence rate of anatomical anal stenosis, including the grade of hemorrhoids, placement of incision, the type of suture, and the experience of surgeon in hemorrhoidectomy. In Table 2, grade 3 and 4 patients for surgery showed no difference. Unfortunately, we were unable to further analyze the same type of suture and experienced surgeon and the placement of the incision which was determined by the location of the hemorrhoid in each patient. In our animal model, we used a transverse incision in rats to simulate a small portion of the PPH wound. This model helped us to exclude the issues related to the placement of the incision, type of suture, and experience of the surgeon. Potentially, we can simulate the grade of hemorrhoids by applying different forces of tension to all small portions.

It seems that performing PPH on large animals like porcine can be difficult due to their limited availability [35]. PPH uses a ring of continuous tiny staples to secure circular incisions, which generates a mechanical force that is oriented horizontally to the incision and focuses on the wound line [36]. This could potentially cause anal stenosis if greater tension is applied during the suturing process. However, it is challenging to verify this through actual rectal surgeries in small animals, so using rat wounds to simulate the impact of tension on scar formation is a viable alternative [37]. Many studies have presented methods capable of exerting horizontal or vertical mechanical forces on wounds, but they produce mechanical forces over a wider area and are not focused on a single line (Table 3). When compressed and fixed at both ends of the incision line, the spreader used in this study generates horizontal tension focused on the incision line, simulating the forces experienced after rectal circular resection and staple fixation in PPH. The primary limitation of this study is that the linear incision on rat skin represented a segment of the circular wound of PPH.

A comprehensive understanding of how tension regulates the cellular contributors of the healing process of wounds is necessary, as healing can be affected by the tension added through surgical methods such as sutures or staples. In this study, we first examined the incidence of complications between conventional hemorrhoidectomy and PPH, and further investigated whether tensile force was involved in the formation of anatomical anal stenosis using a rat model. Anal stenosis is characterized by the narrowing of the anal canal, often resulting from scarring and fibrotic processes [45]. Collagen 1, α-SMA, CTGF, and MMP2 are vital in fibrotic processes. However, these molecular characteristics in anal stenosis remain an area that requires further exploration. Collagen I, a key component of the extracellular matrix, plays multifaceted functional roles in fibrotic processes, contributing to tissue scarring [46], organ fibrosis [47,48], and matrix remodeling [49]. Studies have indicated that fibrotic processes involving collagen deposition can contribute to the development of anal stenosis [50]. Additionally, the mechanical force can shift fibroblasts towards pro-fibrotic phenotypes, leading to myofibroblast differentiation and excessive collagen production driven by ERK-YAP activation [51]. α-SMA is associated with myofibroblast activation, which is linked to tissue fibrosis and contractile activity [52]. Studies have shown that mechanical tension induced by wound splinting can influence α-SMA expression in wounds, affecting myofibroblast differentiation and wound repair [53]. These results could support our finding regarding the elevation of Collagen I and α-SMA expression in the tension group. The expression of CTGF has been associated with fibrotic changes in various tissues, including the kidney, heart, and periodontal tissues [54,55]. MMP-2, a matrix metalloproteinase, along with MMP-9, has been demonstrated to be upregulated in fibrotic conditions, contributing to excessive collagen deposition that results in fibrosis [56]. Additionally, MMP-2 has been linked to angiogenesis and vascular remodeling, exacerbating the progression of fibrosis [57]. The rise of MMP-2 in the tension group might be related to the increased expression of VEGF-A and CD31 proteins. An animal study has shown that there was significant anal stenosis and active angiogenesis in the anorectoplasty and pull-through procedure performed with tension anastomosis. Tension anastomosis resulted in tissue ischemia and was accompanied by the increase in VEGF-C and HIF-1α expression [58]. That might explain the reason for tension-induced angiogenesis. However, the relationship between angiogenesis and anal stenosis remains an area that requires further exploration. Meanwhile, there is existing evidence indicating that excessive angiogenic response has been suggested to promote scar formation [59]. The involvement of angiogenesis in scar formation has been demonstrated in various models, including hypertrophic scars, traumatic brain injury, and myocardial infarction [60,61,62]. The promotion of angiogenesis has been shown to enhance scar formation in specific contexts, such as skin scarring and spinal cord injury [63,64]. Moreover, the downregulation of angiogenic factors like VEGF has been linked to reduced scar formation in hypertrophic scars [65].

Our clinical data showed that PPH significantly improved postoperative pain, whereas it resulted in higher incidences of complications, including anal stenosis, than conventional hemorrhoidectomy. We simulated the status of PPH and developed a rat model to verify PPH’s tension force and found an increase in M1 and M2 macrophages and some cytokines, including IL-1β and IL-10. The cellular contractile forces in the activated myofibroblasts are critical to maintaining scar contracture, and the mean scar area in the tension group was larger than in the control group. Similarly, the distribution of collagen 1, α-SMA, CTGF, and MMP2 deposition in the tension group was significantly broader than in the control group, and the distribution of deposition in the tension group was significantly broader than in the control group. Mechanical tension, such as retraction during a surgery or the use of sutures, may activate fibroblasts and proinflammatory cytokines, ultimately increasing the amount of type I collagen and matrix production. Our data support the notion that exogenous tension leads to a significant increase in proinflammatory factors (M1/M2 macrophages, IL-1β, and IL-10), angiogenic factors (CD31 and VEGF-A), and fibrotic factors (collagen 1, α-SMA, CTGF, and MMP2). Additionally, the anti-fibrotic protein Cyr61 was significantly decreased in the exogenous tension treatment. Some factors, such as IL-6, TGF-β1, and MMP-9, were not significantly changed in the tension group. In addition to the issue of the analytic schedule, a detailed understanding of the tension mechanism is needed.

An imbalance between macrophage M1 and M2 states contributes to the development of a persistent inflammatory microenvironment. Within a chronic inflammatory microenvironment, pro-inflammatory M1 macrophages take on a central role as the primary generators of inflammatory cytokines (such as IL-6 and IL-1β) [66,67,68]. The balance between M1 and M2 macrophages in skin wounds offers valuable insights into determining the age of the wound [69]. During the initial 1 to 7 days after injury, the population of M1 macrophages predominates. Wounds aged between 2 and 5 days consistently exhibit M1/M2 ratios exceeding 2.0. Dr. Wang’s research highlighted distinct distributions of M1 and M2 macrophages in the reparative area of non-traumatic osteonecrosis of the femoral head [70]. Notably, M2 macrophages exhibited accumulation around perivascular regions (with an approximate M1/M2 ratio of 0.05), while M1 macrophages were predominantly present in avascular zones (with an approximate M1/M2 ratio of 12) [69]. In our future studies, we will aim to delve more deeply into the precise distribution and roles of individual M1 and M2 macrophages, as well as the M1/M2 ratio. This will contribute to a more comprehensive understanding of the subject in the future.

The ECM serves a multi-purpose role beyond its scaffold role; it functions as a versatile regulator capable of influencing the activities of extracellular signaling molecules. Additionally, the ECM can directly interact with cell surface receptors, transmitting signals that intricately regulate various cellular functions [71]. Among the array of ECM proteins, a specific subgroup, termed matricellular proteins, exemplified by the CCN family, demonstrates dynamic expression patterns that predominantly fulfill regulatory functions rather than structural ones. The abbreviation CCN stems from the initial three members of this family: CYR61 (CCN1), CTGF (CCN2), and NOV (nephroblastoma overexpressed/CCN3) [72]. Mammalian wound healing and tissue repair exhibit comparable patterns across virtually all organ systems, progressing through three interrelated, yet sequential phases. The process begins with inflammation, transitions into the creation of granulation tissue and the deposition of ECM, and ultimately culminates in the remodeling of the matrix and the resolution of granulation tissue. However, in certain cases, there can be an excessive accumulation of ECM during the wound repair process, which can result in undesirable outcomes such as fibrosis, scarring, and compromised tissue function [25]. Our current data on CTGF and ECM depositions indicated higher amounts of these in the tension group than the control group, supporting the notion that excessive exogenous tension leads to fibrosis, scarring, and loss of tissue function.

It is the goal of wound healing research to determine the conditions for healing with no or minimal scarring. Surgeons may change the placement of incisions to hide scars, either in natural wrinkle lines or in areas that are easily covered and use different sutures to control tension during surgery. Surgeons now focus on minimizing tension through strategic incision placement, as well as taking measures to prevent infection and inflammation, to achieve optimal wound healing and minimal scarring. In closing wounds, it is important to use the appropriate suture technique to reduce tension across the closure. Exploring new methods that can help reduce surgical scarring for the promotion of optimal wound healing, and to minimize scarring is therefore a valuable research direction. Some promising options include tension-offloading topical treatments, laser treatment, dermabrasion devices, and small molecule therapeutics for scarless wound healing.

## 4. Materials and Methods

### 4.1. Study Participants and Patient Flow

This retrospective data collection study was approved by the Institutional Review Board of the Tri-Service General Hospital (TSGHIRB No: 1-108-05-187, issued date 10 December 2019), Taipei, Taiwan, Republic of China. A total of 292 patients underwent hemorrhoidectomy between January 2011 and December 2017. This group was divided into conventional hemorrhoidectomy (open, closed, and mixed, 125 patients) and PPH (procedure of prolapse and hemorrhoids, 167 patients). Sample size was calculated using Epitools (https://epitools.ausvet.com.au/) (accessed on 20 May 2023). For a case-control study with a presumed 3% complication rate in the control group, an odds ratio of 6, 95% confidence level, and 80% power, the minimum sample size was estimated as 80 per group. Among the 167 PPH cases, 5 patients had a history of anal surgery, 5 patients had a psychiatric history, 3 patients were lost to follow-up, 61 patients underwent an additional hemorrhoidectomy, and 18 patients received an additional cut in stapler line, so were excluded from the analysis. Among those who underwent a conventional hemorrhoidectomy, 15 patients had a history of anal surgery, 2 patients had a psychiatric history, 3 patients received non-intravenous general anesthesia, 3 patients were lost to follow-up, 2 patients had 100% CWA (circumferential wound of the anus), and 1 patient was classed as grade II, and were excluded from the analysis. Finally, a total of 174 eligible patients who underwent either conventional hemorrhoidectomy (99) or pure PPH (75) were analyzed.

### 4.2. An Animal Model for the Study of Tensile Force

All animal experimental procedures were reviewed and approved by the Experimental Animal Committee of the National Defense Medical Center (Application Form No. IACUC-19-132, issued 26 April 2019). Fourteen two-month-old healthy Sprague Dawley rats were used for this experiment, animals were presumed from BioLASCO (Taipei, Taiwan). The animals were housed in the animal center for one week as an acclimatization period before commencing the study. To minimize potential confounders, we marked the number on the tail of the rats and the cage cards. The Sprague Dawley rats were anesthetized with 20 mg/kg Zoletil mixed with 5 mg/kg Rompun intramuscularly, and then we shaved and disinfected the skin. Two incision wounds of 1 cm in length were placed on the skin of the back of the rats. The incision wound was sutured with a 4-O suture. In order to reduce individual differences, the wounds of the experimental group and the control group were established simultaneously in the same experimental rat. The wound in the experimental group (tension group) was on the upper back, and the spreader was set in parallel to the wound with a fixed tension, while the wound in the control group was on the lower back without any tension. The spreader was compressed in a direction parallel to the wound and fixed at both ends of the wound. When the spreader was pressed to a distance of 2 cm at both ends, it could produce a tension of about 85 g weight. Ketoprofen was given twice daily at a dosage of 2.5 mg/kg for analgesic purposes. We carefully observed the changes in the wound and ensured the position and distance of the spreader were accurate; if the distance was greater than 2.2 cm, an adjustment was required to maintain the distance of 2 cm. Photographed records were taken on the 3rd, 7th, 10th, and 12th days after the incision. The animals were sacrificed on the 3rd, 7th, and 12th days after surgery (3 for the 3rd and 7th days, 8 for the 12th days, respectively). To determine the expression of biomarkers related to inflammation, angiogenesis, and scar formation, the skin tissues were fixed with 4% paraformaldehyde, embedded in paraffin, and 5 μm slides were prepared for histological analysis. Skin samples were excluded if they exhibited signs of infection or have been bitten by rats, and there were no exclusions in this study. This work has been reported in accordance with the ARRIVE (Animals in Research: Reporting In Vivo Experiments) guidelines [73].

### 4.3. Hematoxylin-Eosin (H&E) Staining

On the 12th day after the incision, eight mice were sacrificed to collect scar tissue for H&E and Masson’s trichrome staining. H&E staining was performed to observe the area of granulation tissue and inflammatory cell infiltration. Under the microscope, tissue sections can be used to identify areas exhibiting characteristics of granulation tissue, which is composed of fibroblasts, collagen fibers, and newly formed blood vessels. The criteria we employed to evaluate inflammatory cell infiltration involved identifying inflammatory cells and quantifying the distribution area of these cells. Inflammatory cells typically appear as small, darkly stained nuclei surrounded by a pale-staining cytoplasm in H&E staining. The tissue sections were observed under a microscope and images were captured using a digital camera to delineate the distribution area of inflammatory cells. The epidermis is the outermost layer of the skin, typically characterized by a dark purple stratified arrangement of distinct layers from the stratum basale to the stratum corneum.

### 4.4. Masson’s Trichrome Staining

Masson’s trichrome staining was used to observe the deposition of collagen fibers and determine the scar area. The scar tissue sections were subjected to staining using hematoxylin and eosin solution (obtained from Merck Millipore, Darmstadt, Germany), as well as Masson’s trichrome stain kit (procured from ScyTek Laboratories, Logan, UT, USA). These staining procedures were performed following the guidelines provided by the respective manufacturers. The stained sections were photographed using a light microscope (ZEISS, Oberkochen, Germany). The scar area was determined by Zen software, version 2.6, and the area percentage of collagen was analyzed by Image J, version 1.44a (http://imagej.nih.gov/ij/) (accessed on 1 August 2023). The deposition levels of collagen were present as a percentage of area (% of area), which refers to the proportion of the total area of a tissue sample occupied by staining for collagen.

### 4.5. Immunohistochemistry Staining

The 5 μm tissue sections were deparaffinized with xylene (Ferak Berlin, Germany) and rehydrated gradually. After repairing the antigen and quenching endogenous peroxidase, the sections were blocked with 10% goat serum in phosphate-buffered saline (PBS) for 1 h and then incubated with the primary antibody. The primary antibodies used for immunohistochemistry, including Col1, α-SMA, and Cry61, were obtained from GeneTex (Irvine, CA, USA); IL-1β, IL-6, IL-10, and CD31 were purchased from Cell Signaling Technology (Beverly, MA, USA); and TGF-β1, CTGF, MMP-2, and MMP-9 were bought from Santa Cruz Biotechnology Inc. (Dallas, TX, USA). All the sections were counterstained with hematoxylin solution and photographed under a light microscope. ImageJ software was used to determine the protein expression in the granulation tissue or scar region. The protein expression levels of α-SMA were present as a percentage of area (% of area), which refers to the proportion of the area of the tissue section being analyzed of a tissue sample occupied by staining for the α-SMA. Cytokine protein expression levels were present as an integrated density of staining within a defined area (IntDen/Area) or integrated density, representing the sum of the gray values of all the pixels in the defined area of interest. In IHC, this typically corresponds to the staining intensity, indicating the amount of target antigen present in the tissue sample.

### 4.6. Immunofluorescence Staining

To examine the distribution of M1 and M2 macrophages in the wound tissue, 20 μm frozen tissue sections were first treated by blocking with 0.1% normal goat serum for 30 min. Subsequently, these sections were subjected to incubation with antibodies against F4/80, CD206 (GeneTex, Irvine, CA, USA), or NOS2 antibody (Santa Cruz Biotechnology, Inc., Dallas, TX, USA) in a 1:200 dilution with blocking solution for 2 h. Following a wash with PBS, the sections underwent a secondary antibody incubation step (using goat anti-mouse IgG H&L Alexa Fluor 488 and Alexa Fluor 594 antibodies from Invitrogen, Waltham, MA, USA) at a dilution of 1:1000 for 1 h. Cell nuclei were counterstained with DAPI in a 1:5000 dilution. Finally, the samples were mounted using Permount Mounting Medium (SP15-500, Fisher Scientific, Waltham, MA, USA) and subjected to imaging using a light microscope (ZEISS, Oberkochen, Germany).

### 4.7. Statistical Analyses

Descriptive statistics, including percentages and frequencies, were used for categorical variables, while means and standard deviations (SD) were presented for continuous variables. The Chi-squared test was performed for comparisons of categorical variables in two groups. The independent sample t-test was used to compare continuous variables of the baseline characteristics of these cohorts and histological quantified data from the animal study. Multiple logistic regression analysis models were implemented to identify independent risk factors for overall complications after the operation. The odds ratio (OR) and 95% confidence interval (CI) were provided. All analyses were performed using the SPSS version 19.0 statistical package (IBM, Armonk, NY, USA). A *p*-value less than 0.05 is considered statistically significant.

## 5. Conclusions

Our clinical data showed that PPH significantly improved postoperative pain, but that it resulted in higher incidences of complications, including anal stenosis, than hemorrhoidectomy. We simulated the status of PPH and developed a rat model to verify PPH’s tension force, including the scarring area and the deposition of proinflammatory factors, angiogenic factors, and fibrotic factors, using H&E, Masson’s trichrome, immunohistochemistry, and immunofluorescence staining. Based on the findings of our animal model, the development of a lesser tensile force for PPH to decrease the deposition of proinflammatory factors, angiogenic factors, and fibrotic factors is urgently required.

## Figures and Tables

**Table 1:** Demographics.

	Hemorrhoidectomy	PPH	*p*-Value
n	99	75	
Gender			0.962
Male (%)	53 (53.5)	39 (52.0)	
Female (%)	46 (46.5)	36 (48.0)	
Age (mean (SD))	51.87 (15.95)	51.01 (12.28)	0.697
Grade 4 (%)	66 (66.7)	47 (62.7)	0.699
3	33 (33.3)	28 (37.3)	
4	66 (66.7)	47 (62.7)	
ASA (%)			0.338
1	34 (34.3)	34 (45.3)	
2	52 (52.5)	33 (44.0)	
3	13 (13.1)	8 (10.7)	
Operation Time (mean (SD))	51.72 (17.85)	41.60 (13.59)	<0.001 *
Length of stay (LOS) (mean (SD))	1.44 (0.66)	1.51 (0.67)	0.54
Constipation History			0.727
Yes	50 (50.5)	35 (46.7)	
No	49 (49.5)	40 (53.3)	
Complications (%)	3 (3.0)	8 (12.5)	0.042 *
Anatomical anal stenosis (%)	0 (0.0)	5 (6.7)	0.032 *
Bleeding (%)	0 (0.0)	1 (1.3)	0.889
Urine retention (%)	3 (3.0)	2 (2.7)	1
Painkiller in 1st OPD (%)			0.002 *
Yes	41 (41.4)	14 (18.7)	
No	58 (58.6)	61 (81.3)	
Recurrence (%)			0.808
Yes	1 (1.0)	2 (2.7)	
No	98 (99.0)	73 (97.3)	

* *p*-value < 0.05 was considered statistically significant after test.

**Table 2:** Risk analysis.

	Odds Ratio	95% CI			*p*-Value
Surgery					
Hemorroidectomy	1.0 (reference)				
PPH	5.913	4.903	~	40.541	0.049 *
Sex					
Male	1.0 (reference)				
Female	0.667	0.506	~	3.429	0.577
Age	1.009	0.062	~	2.084	0.774
Grade					
3	1.0 (reference)				
4	2.174	1.740	~	13.056	0.344
ASA	0.532	0.380	~	2.398	0.324
operation time	1.014	0.049	~	2.079	0.581
Length of stay (LOS)	2.827	1.615	~	9.420	0.016 *
Constipation History					
No	1.0 (reference)				
Yes	2.598	2.003	~	13.948	0.204

* *p*-value < 0.05 was considered statistically significant after test.

**Table 3:** Summary of studies using horizontal or vertical mechanical forces on wounds in rat or mice models.

Reference	Animal Species	Primary Mechanism	Advantages	Limitations
Present study	Rat	A spreader compressed and fixed at both ends of the incision line and generated horizontal tension focused on the incision line (85-g force).	Less harmful to the surrounding skin	A transverse incision in rats is to simulate a small portion of the PPH wound.
Cremers et al. [38] Son et al. [39]	Mice	Splinted the edges of full-thickness wounds. (The mechanical force is not stated)	A historical, robust, and reliable model to study myofibroblast biology	Harmful to the surrounding skin.
JIMI et al. [40]	Mice	The abdominal muscle wall may be potentially/physiologically under longitudinal static tension to some extent (1.8 mN, 1.84-g force).	Under physiological conditions without using any artificial factors	The tension that can be formed is weak, easily affected by animal activities, and has large variations.
Aarabi et al. [41] Wang et al. [42]	Mice	Biomechanical loading device engineered from expansion screws (1.5–2.7 N/mm^2^, 153–275-g force).	Capable of producing horizontal or vertical mechanical force stably	The device affects the animals’ daily activities and causes discomfort.
Chin et al. [43]	Mice	A servo-controlled skin-stretching device to produce predetermined tension in mice (35–90-g force).	Can achieve cyclically or static stretch.	Must be operated under anesthesia and cannot be used for long periods of time.
Pickett et al. [44]	Rat	Excise some skin between incisions to produce closing tensions (70–120-g force).	Without using any artificial factors	Skin loss up to 50–60 mm widths.

## Data Availability

Data associated with the publication are available upon request by the corresponding author. The data are not publicly available due to ethical reasons.
